# Abdominal Cæsarean Section for Eclampsia

**Published:** 1914-12-12

**Authors:** 


### ABDOMINAL CÆSAREAN SECTION FOR ECLAMPSIA.

When abdominal Cæsarean section had been rendered a comparatively safe and simple operation, the idea naturally arose among those who believed that the proper treatment of puerperal convulsions includes the delivery of the fœtus at the earliest possible moment, that this operation might prove to have a definite place in the treatment of eclampsia. A very large number of such abdominal deliveries have taken place, and 500 of them have been tabulated by Dr. Peterson in the *American Journal of Obstetrics and Gynæcology.* He communicated with leading obstetric surgeons in all civilised countries, asking for information about all such cases in their own practice, whether already published or not; and asking also for the operator's name of any other such cases that they might be cognisant of. When 500 cases had been thus accumulated, the polls were closed, and the statistics analysed. The cases were in the care of 259 separate operators, and the majority of them, naturally enough, were in the United States; in fact, Dr. Peterson got wind of only four cases in England, and the rest of the Empire was represented even more meagrely. For statistical purposes he separates the cases in the period 1908-13 from those previously occurring, on the ground that the imperfect technique of the earlier years would give an incorrect impression of the success or failure of the treatment at the present time. Up to 1908 the maternal mortality of eclampsia treated by abdominal Cæsarean section was 48 per cent.; since then it has been under 26 per cent. Even this figure can be regarded as much in excess of what it need be if infection *per vaginam* can be excluded, or operation avoided when there is any reason to suspect it. Dr. Peterson finds that convulsions ceased after abdominal delivery in 55 per cent. of his cases: this is practically the same proportion as in delivery *per vaginam*, naturally or artificially. Even when the convulsions do thus cease, nearly 20 per cent. of the patients die after abdominal Cæsarean section: when the convulsions do not cease, the mortality rises to 31 per cent. As evidence of the value of the method, it is shown that in sixty cases in which five or less convulsions had occurred, in which at the most two vaginal examinations had been made, and no attempts whatever at delivery from below, the maternal mortality was but 15 per cent. Delay means increase in the mortality. In cases having had six or more convulsions it was 30 per cent. Excluding premature children, the fœtal mortality of this method of treatment was only 3.6 per cent.; or 10.7 per cent. if all children dying in the first three days are counted in. In this series 83.75 per cent. of the mothers were primiparæ; this frequency is in a sense fictitious, because the longer duration of primiparous deliveries and the greater difficulty of rapid artificial delivery *per vaginam* naturally resulted in an unduly high proportion of Cæsarean sections in primiparous patients. It is curious, however, that the mortality among the latter was actually less than among the multiparæ. Much emphasis is laid upon the influence of sepsis upon the statistics; attempts to deliver, and even vaginal examinations, are deprecated altogether. Dr. Peterson concludes that in the present state of knowledge older and more tried methods of emptying the uterus in eclampsia give better results when the pelvis and soft parts are normal, and that they should not lightly be discarded in favour of the more brilliant and easier Cæsarean operation. But the latter, he affirms, has now reached a stage at which it can no longer be disregarded by obstetricians; and he clearly looks forward to a time when Cæsarean section will be recognised as proper treatment and employed under most favourable auspices without any risks having been added to it by contamination of the passages from below.

